# New congenital coronary artery anomaly – double supply of single left anterior descending coronary artery from the left and right coronary sinuses: a case report

**DOI:** 10.1186/s13256-016-1003-7

**Published:** 2016-08-02

**Authors:** Yunis Daralammouri, Malik Ghannam, Bernward Lauer

**Affiliations:** 1 Department of Cardiology, Al Najah National University Hospital, Nablus, West Bank, Palestine; 2 Department of Cardiology, Zentralklinik Bad Berka, Robert-Koch-Allee 9, 99437 Bad Berka, Germany

**Keywords:** Congenital coronary anomaly, Dual left anterior descending artery distribution

## Abstract

**Background:**

A normal anatomy of coronary arteries is important to have adequate cardiac muscle blood supply especially during extraneous physical activities. This case report describes a rare coronary anomaly in which the accessory coronary artery arose from the right coronary artery, reentered the left anterior descending coronary artery, and then ran as a single vessel.

**Case presentation:**

We present a case of a coronary anomaly in a 47-year-old white man who presented with atypical angina. Computed tomographic angiography and coronary angiography showed a variant of dual left anterior descending coronary artery not previously described. Our patient’s accessory coronary artery arose from his right coronary artery. It took an intramuscular course beneath the right ventricular outflow tract in the interventricular septal area to the anterior interventricular sulcus, giving off septal perforators that reentered his medial left anterior descending coronary artery. Both vessels ran after the anastomosis in the anterior interventricular sulcus as a single vessel.

**Conclusions:**

We propose that this anomaly represents a new variant of coronary artery anomaly. This coronary artery anomaly does not cause ischemia. Recognition of this coronary anomaly is important in patients undergoing percutaneous coronary intervention or coronary artery bypass graft operations.

## Background

A normal anatomy of coronary arteries is important to have adequate cardiac muscle blood supply especially during extraneous physical activities. Congenital coronary anomalies (CAA) are rare and reportedly occur in 0.64 to 1.3 % of patients undergoing coronary angiography. Approximately 80 % of these coronary anomalies are benign; only 20 % cause symptoms [1]. Dual left anterior descending coronary artery (LAD) has been reported to occur with an incidence of 1 % [2, 3], and most of the patients were asymptomatic [3].

This case report describes a rare variation, which has not been reported in the literature, in which the accessory coronary artery arose from the right coronary artery (RCA), reentered the LAD, and then ran as a single vessel.

## Case presentation

**Fig. 1:**
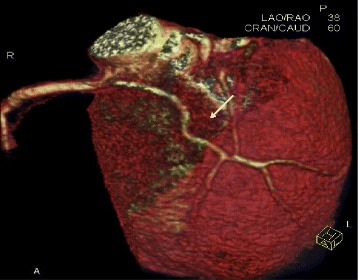
Normal electrocardiogram-gated multidetector row computed tomography findings. The lateral oblique volume-rendered image shows an accessory coronary artery arising from the proximal right coronary artery. It took an intramuscular course beneath the right ventricular outflow tract (removed with manual editing) and reentered the medial left anterior descending coronary artery (*arrow*)

**Fig. 2:**
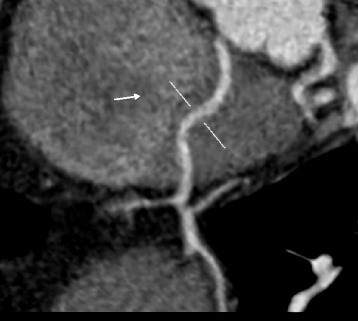
Multiplanar reconstruction computed tomography image demonstrates the intramuscular course of the accessory coronary artery (*arrow*)

**Fig. 3:**
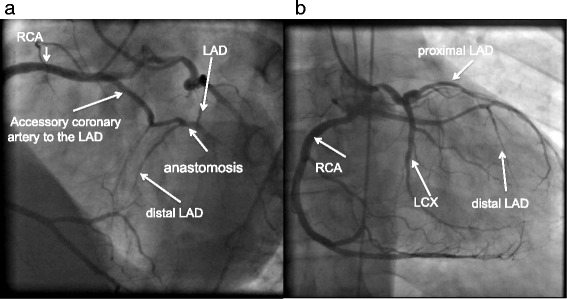
Simultaneous coronary angiogram of the right coronary artery and left circulation showing an anomalous accessory coronary artery arising from the proximal right coronary artery and reentering the proper left anterior descending coronary artery; the left circumflex artery is normal. **a** Left anterior oblique caudal view. **b** Anteroposterior view. *LAD* left anterior descending coronary artery, *LCX* left circumflex artery, *RCA* right coronary artery

Here we present a case of a rare coronary anomaly in a 47-year-old white man with an unremarkable past medical history who presented with atypical angina in late 2012; he does not have a family history of any heart disease, he is not allergic to anything, and he has never had any cardiac procedure. A physical examination and resting electrocardiogram results were normal, and transthoracic echocardiography showed no wall-motion abnormality and normal global left ventricular systolic function. His biochemical parameters were within normal limits. The treadmill exercise test showed 1-mm down-sloping ST-segment depression in leads V4 to V6. Multidetector computed tomographic angiography was performed to evaluate coronary artery disease and revealed an accessory coronary artery arising from the proximal RCA. It took an intramuscular course between the right ventricular outflow tract in the interventricular septal area to the anterior interventricular sulcus (AIVS), giving off septal perforators that reentered the medial LAD. Both vessels ran after the anastomosis in the AIVS as a single vessel (Figs. 1 and 2). A coronary angiography was performed to characterize the coronary anomaly and to detect any resultant ischemia. His RCA was dominant, giving origin proximally to an anomalous accessory coronary artery that reentered his medial LAD and ran as a single vessel (Fig. 3). The left main artery arose from the left coronary sinus and branched into a proper LAD and a circumflex artery (Fig. 2). There was no significant obstructive coronary artery disease. We measured the pressure-derived fractional flow reserve (FFR) to detect ischemia in the distal LAD after the anastomosis of the accessory coronary artery using a 0.014-inch (0.3556 mm) pressure wire (Volcano Corporation) during infusion of increasing doses of dobutamine (5, 10, 20, and 30 μg kg^−1^ minute^−1^, each in 5-minute increments). The FFR was >0.95. Thus, this anomaly did not cause ischemia during exercise. It was decided that his chest pain might be musculoskeletal in origin; therefore, he was given non-steroidal anti-inflammatory medication, after 1 month of follow up visits his pain was gone.

## Discussion

Dual LAD can be associated with congenital heart disease such as tetralogy of Fallot and complete transposition of the great arteries, where it has surgical importance at the time of corrective surgery [4]. Spindola-Franco *et al*. classified dual LAD into four angiographic subtypes based on the origin and course of both LADs as follows [3]. Type I: Running in the AIVS, the short LAD is generally the source of all major proximal septal perforators. The long LAD also runs in the AIVS, descending on the left ventricular side of the AIVS, and then reentering the distal AIVS. It is the source of the major diagonal vessels. Type II: The short LAD is the same as in Type I. The long LAD differs only in that it descends on the right ventricular side, rather than the left, before reentering the AIVS. Type III: The long LAD travels intramyocardially in the ventricular septum. The short LAD is the same as in Types I and II. Type IV: The long LAD originates from the RCA and enters the anterior interventricular groove. Type IV dual LAD is a rare congenital anomaly of the coronary arteries and may be mistreated. The exact description of coronary artery anatomy is important in patients undergoing coronary artery bypass graft operations or percutaneous coronary intervention [5]. In our case, the accessory coronary artery arose from the RCA and took a course between the right ventricular outflow and aorta, then took an intramuscular course beneath the right ventricular outflow tract in the interventricular septal area to the AIVS. It gave off septal perforators, then reentered the medial LAD, and both vessels ran after the anastomosis in the AIVS as a single vessel. This course can be associated with limiting coronary blood flow, when dilation of the aorta occurs during exercise or due to the stretch of the intramural segment. The FFR measurements during the infusion of increasing doses of dobutamine showed that this coronary anomaly does not lead to ischemia. We propose that this anomaly represents a new type of dual LAD.

## Conclusions

Our case, in which the accessory coronary artery arose from the RCA, reentered the LAD, and then ran as a single vessel, is the first such report in the literature. This coronary artery anomaly does not cause ischemia. Recognition of this coronary anomaly is important in patients undergoing percutaneous coronary intervention or coronary artery bypass graft operations.

## Abbreviations

AIVS, anterior interventricular sulcus; CAA, congenital coronary anomalies; FFR, fractional flow reserve; LAD, left anterior descending coronary artery; RCA, right coronary artery
